# Multidimensional fatigue inventory and post-polio syndrome – a Rasch analysis

**DOI:** 10.1186/s12955-015-0213-9

**Published:** 2015-02-12

**Authors:** Anna Dencker, Katharina S Sunnerhagen, Charles Taft, Åsa Lundgren-Nilsson

**Affiliations:** Centre for Person-centred Care, Institute of Health and Care Sciences, Sahlgrenska Academy, University of Gothenburg, Gothenburg, Sweden; Department of Clinical Neuroscience and Rehabilitation, Institute of Neuroscience and Physiology, Sahlgrenska Academy, University of Gothenburg, Gothenburg, Sweden; Institute of Health and Care Sciences, Sahlgrenska Academy, University of Gothenburg, Box 457, 405 30 Gothenburg, Sweden

**Keywords:** Fatigue, MFI-20, Post-polio, Rasch analysis

## Abstract

**Background:**

Fatigue is a common symptom in post-polio syndrome (PPS) and can have a substantial impact on patients. There is a need for validated questionnaires to assess fatigue in PPS for use in clinical practice and research. The aim with this study was to assess the validity and reliability of the Swedish version of Multidimensional Fatigue Inventory (MFI-20) in patients with PPS using the Rasch model.

**Methods:**

A total of 231 patients diagnosed with PPS completed the Swedish MFI-20 questionnaire at post-polio out-patient clinics in Sweden. The mean age of participants was 62 years and 61% were females. Data were tested against assumptions of the Rasch measurement model (i.e. unidimensionality of the scale, good item fit, independency of items and absence of differential item functioning). Reliability was tested with the person separation index (PSI). A transformation of the ordinal total scale scores into an interval scale for use in parametric analysis was performed. Dummy cases with minimum and maximum scoring were used for the transformation table to achieve interval scores between 20 and 100, which are comprehensive limits for the MFI-20 scale.

**Results:**

An initial Rasch analysis of the full scale with 20 items showed misfit to the Rasch model (p < 0.001). Seven items showed slightly disordered thresholds and person estimates were not significantly improved by rescoring items. Analysis of MFI-20 scale with the 5 MFI-20 subscales as testlets showed good fit with a non-significant *x*^2^ value (p = 0.089). PSI for the testlet solution was 0.86. Local dependency was present in all subscales and fit to the Rasch model was solved with testlets within each subscale. PSI ranged from 0.52 to 0.82 in the subscales.

**Conclusions:**

This study shows that the Swedish MFI-20 total scale and subscale scores yield valid and reliable measures of fatigue in persons with post-polio syndrome. The Rasch transformed total scores can be used for parametric statistical analyses in future clinical studies.

## Background

After recovery from acute poliomyelitis, many persons experience symptoms again after a stable period of 15 years or longer. This condition is known as post-polio syndrome (PPS). PPS symptoms are progressive and include muscle weakness, atrophy, cold intolerance, pain and extensive fatigue. Incidence rates of between 15 and 80% have been reported [1]. The causes of PPS symptoms are still not completely understood. Fatigue is commonly reported in PPS and many patients report that fatigue is the most disabling symptom impacting on their health-related quality of life [2-5]. Earlier research has described fatigue in PPS as a multidimensional construct comprising mental, emotional and physical aspects [6], where physical fatigue is most common [2]. Both general and muscular fatigue is common in PPS and can be mixed with other symptoms, such as weakness [2,3] and deconditioning. The possible impact of central fatigue [7] has been discussed but recently this has been questioned [8].

There is a need for validated fatigue questionnaires for use in clinical practice and research. One of the most widely used instruments for assessing multiple dimensions of fatigue is the 20 item Multidimensional Fatigue Inventory (MFI-20), measuring five dimensions of fatigue. The MFI-20 was developed and psychometrically evaluated within the framework of classical test theory. Modern test theory with Rasch analysis provides additional and more detailed psychometric information regarding the dimensionality of the scale, validity and appropriateness of summated scores, response categories, targeting of the scale, and item bias, i.e. differential item functioning (DIF). Moreover, Rasch analysis formally tests requirements needed to transform ordinal scales, such as the MFI-20 [9,10], into interval level measurements [11].

### Aim

The aim was to assess the validity and reliability of the MFI-20 in patients with post-polio syndrome using the Rasch model.

## Methods

### Participants and setting

Patient data were obtained from two clinical studies. The first study [12] was conducted between 2002 and 2003 at post-polio out-patient clinics located at four major Swedish university hospitals: Danderyd University Hospital (n = 47), Huddinge University Hospital (n = 41), University Hospital in Uppsala (n = 29) and Sahlgrenska University Hospital in Gothenburg (n = 26). All patients (n = 143) answered the MFI-20. The second study included all post-polio patients booked for a first visit at the Polio Clinic, Rehabilitation Medicine at Sahlgrenska University Hospital between 2002 and 2012. In total, 88 of 98 patients answered the MFI-20 (n = 88). All patients were examined by a rehabilitation medicine physician. After history and confirmed diagnosis (including electromyography) PPS was diagnosed according to the definitions of the March of Dimes [13]. Ethical approval was obtained for both data collections and patients gave written informed consent before inclusion into the studies. Data from the two studies were anonymized before being transferred for use in the present study.

### MFI-20

The Swedish version of the Multidimensional Fatigue Inventory (MFI-20) was used [14]. MFI-20 is a 20-item self-administered questionnaire designed to measure fatigue in five four-item subscales: General fatigue, Physical fatigue, Reduced activity, Reduced motivation and Mental fatigue [9,10]. MFI-20 has an even proportion of positively and negatively worded items that are rated on a 5-point Likert scale. Subscale scores (range 4–20) are calculated as the sum of item ratings and a total fatigue score (range 20–100) is calculated as the sum of subscale scores. Higher scores indicate a higher level of fatigue. Psychometric validation of MFI-20 has shown good validity and reliability [15-17]. MFI-20 is validated in Sweden in patients with cancer, fibromyalgia and chronic widespread pain [14,18-20].

### Statistical analyses

SPSS (Statistical Package for Social Services Version 20 (SPSS Inc., Chicago, IL, USA) was used for descriptive statistics. Psychometric testing was performed with Rasch Unidimensional Measurement Models computer software (RUMM 2030) [21].

### Rasch analysis

Rasch analysis was used to test if the data conformed to the assumptions of the Rasch measurement model, i.e. stochastic ordering of items, local response independency, and unidimensionality [22-24].

Category structures of response alternatives were examined to make sure that they are used in a consistent manner by the respondents. Thresholds are the points where the probability of choosing one response category is equal to the probability of choosing an adjacent category. Disordering of thresholds might mirror problems for the respondents to answer to the item and might need rescoring by collapsing response options [25].

Fit of items and persons were evaluated with item-trait interaction with standardised mean person and item fit. The standardized mean values of the person and item fit residual by a mean (SD) score of 0.0 ± 1.0 specifies a good fit. A *x*^2^ statistic was used to assess the invariance of the ordering of items at different levels of perceived fatigue. A non-significant *x*^2^ indicates that the hierarchical ordering of items remains the same at different levels of the underlying trait. A non-significant *x*^2^ probability value of > 0.05 together with standardized fit residuals (differences between observed and expected values) between −2.5 and +2.5 indicate adequate fit of individual person and item residuals. A Bonferroni adjustment was used to adjust for multiplicity [26].

The Rasch model implies local independence of items to confirm unidimensionality. To test the assumption of unidimensionality, principal components analysis (PCA) was performed on standardized residuals and was used to examine the correlation between the items and the residuals. When the Rasch factor is extracted no pattern should remain in the residuals. Person estimates of the items with the most positive and negative residuals were then compared by means of paired sample t-tests for the difference between persons. The lower confidence interval for the number of significant tests should not exceed 5% [27]. Local dependency is considered present when the correlation of the residuals is over 0.3, in which case response dependent items can be combined and tested together in a testlet [25].

Differential Item Functioning (DIF) was tested between groups of different gender (women and men) and age (dichotomized as under and over 63 years) using ANOVA. DIF is a form of measurement bias and refers to differences in the probability of giving a certain response between groups [28,29].

Targeting of the scale was illustrated by means of person-item threshold distribution plots where the centre of the scale, zero, denotes average difficulty of items. For a well-targeted scale the mean value of person ability should be zero [25].

Reliability of the scale was computed with person separation index (PSI). PSI is equivalent to Cronbach’s alpha and should be >0.70 for group use and >0.85 for individual use [25].

Fit to the Rasch model allows for a transformation of scores, where the raw scores consisting of ordinal data can be converted into a logit interval scale and transformed into a metric estimate score for use in parametric statistical analyses within the same range as the original MFI-20 scores [25].

## Results

A total of 231 patients diagnosed with post-polio syndrome completed the MFI-20 questionnaire. The mean age of participants was 62 years and 61% were females (Table 1). Median and range of MFI-20 scores are reported in Table 2. Missing value rates were low (0.4-1.3%).

**Table 1:** **Demographics of study group, n = 231**

	**N (%)**
*Gender*	
Women	141 (61.0)
Men	90 (39.0)
*Age, years*	
<55	48 (20.8)
55-62	65 (28.1)
63-70	67 (29.0)
>70	51 (22.1)

**Table 2:** **Descriptive statistics of MFI-20 scores, n = 231**

	**Median**	**Range**
General fatigue	15	4 – 20
Physical fatigue	15	4 – 20
Reduced activity	13	4 – 20
Reduced motivation	8	4 – 20
Mental fatigue	9	4 – 20
Total score		20 – 100

### Initial fit and disordered thresholds

An initial Rasch analysis of all items in the five subscales showed misfit to the Rasch model with significant *x*^2^ value (p < 0.001) for item-trait interaction. Both person and item fit showed high fit residuals (2.17 vs 1.53). Fit indices for the separate analyses, including person and item fit residual means and SDs along with ideal values, are shown in Table 3. Seven of 20 items displayed disordered thresholds. Six of these (#3, I feel very active; #5, I feel tired; #7, I keep my thoughts on things; #9, I dread having to do things; #14, Physically I am in bad condition; # 20, Physically I am in excellent condition) showed only slightly disordered thresholds and no misfit (fit residual < 2.5). Hence these were not rescored [30]. One item (#19, My thoughts easily wander) showed both disordered thresholds and misfit with fit residual over 2.5. To explore if rescoring this one item would be necessary all person estimates with item #19 rescored (0,1,1,1,2) were compared to the person estimates without rescoring [31]. The difference between rescored and original person estimates was not significant (paired samples t-test, p = 0.138). Therefore, no item was rescored in subsequent analyses.

**Table 3:** **Fit of the MFI-20 to the Rasch model**

	**Item residual**		**Person residual**		***x*** ^**2**^		**Unidimensionality**		
**Analysis name**	**Mean**	**± SD**	**Mean**	**± SD**	**Value**	**p**	**PSI**	**test %**	**(LCI) %**
MFI-20 20 items	0.50	2.17	−0.26	1.53	176.42	0.00	0.92	17.4	14.6
MFI-20 **5 testlets**	**0.25**	**1.51**	**−0.31**	**1.02**	**22.80**	**0.09**	**0.86**	**4.4**	**1.6**
General fatigue (GF)	0.14	1.06	−0.45	1.10	31.19	0.00	0.69	2.5	−0.5
**GF 2 testlets**	**0.22**	**0.19**	**−0.41**	**0.69**	**4.01**	**0.68**	**0.77**	**1.5**	**−1.5**
Physical fatigue (PF)	−0.03	1.81	−0.46	1.06	25.02	0.01	0.73	0.5	−2.5
**PF 2 testlets**	**0.26**	**0.21**	**−0.57**	**0.90**	**7.35**	**0.29**	**0.80**	**3.1**	**0.0**
Reduced activity (RA)	0.28	1.69	−0.50	1.17	24.63	0.02	0.75	3.8	0.8
**RA 2 testlets**	**0.30**	**0.07**	**−0.48**	**0.73**	**3.39**	**0.76**	**0.82**	**5.1**	**2.2**
Reduced motivation (RM)	−0.02	1.29	−0.38	1.16	25.62	0.01	0.30	1.5	−1.5
**RM 2 testlets**	**0.18**	**0.42**	**−0.42**	**0.71**	**7.11**	**0.31**	**0.52**	**1.0**	**−2.0**
Mental fatigue (MF)	0.26	1.42	−0.40	1.14	19.46	0.08	0.66	3.9	0.9
**MF 2 testlets**	**0.33**	**0.02**	**−0.67**	**1.12**	**8.15**	**0.23**	**0.74**	**1.5**	**−1.5**
***Ideal Values***	***0.0***	***<1.4*** ^***a***^	***0.0***	***<1.4***		***>0.05*** ^***b***^	***>0.85***		***(LCI <5%)***

^a^May be higher when unequal length testlets present b) Bonferroni adjusted.

PSI = Person Separation Index, SD = Standard Deviation, CI = Confidence Interval.

No differential item functioning (DIF) was shown in relation to gender (women and men) or age (dichotomized as under and over 63 years).

### Resolving fit to the Rasch model

To deal with response dependency of items found in all subscales a testlet analysis with the five dimensions as testlets was performed. This subtest with the five subscales as testlets showed good fit with non-significant *x*^2^ value (p = 0.089), see Table 3. After performing PCA on the residuals comparisons of the subsets based on positively loaded versus negatively loaded subsets were performed. The independent t-tests between those groups indicated satisfactory unidimensionality of the scale evidenced by the lower confidence interval for the number of significant t-tests overlapping 5% (number of significant t-tests was 4.4%, 95% CI 1.6 – 7.3). Reliability as expressed by the person separation index (PSI) was good (PSI = 0.86) for the testlet solution, but somewhat lower than the initial analysis of the twenty items in the MFI-20 (PSI 0.92).

**Figure 1:**
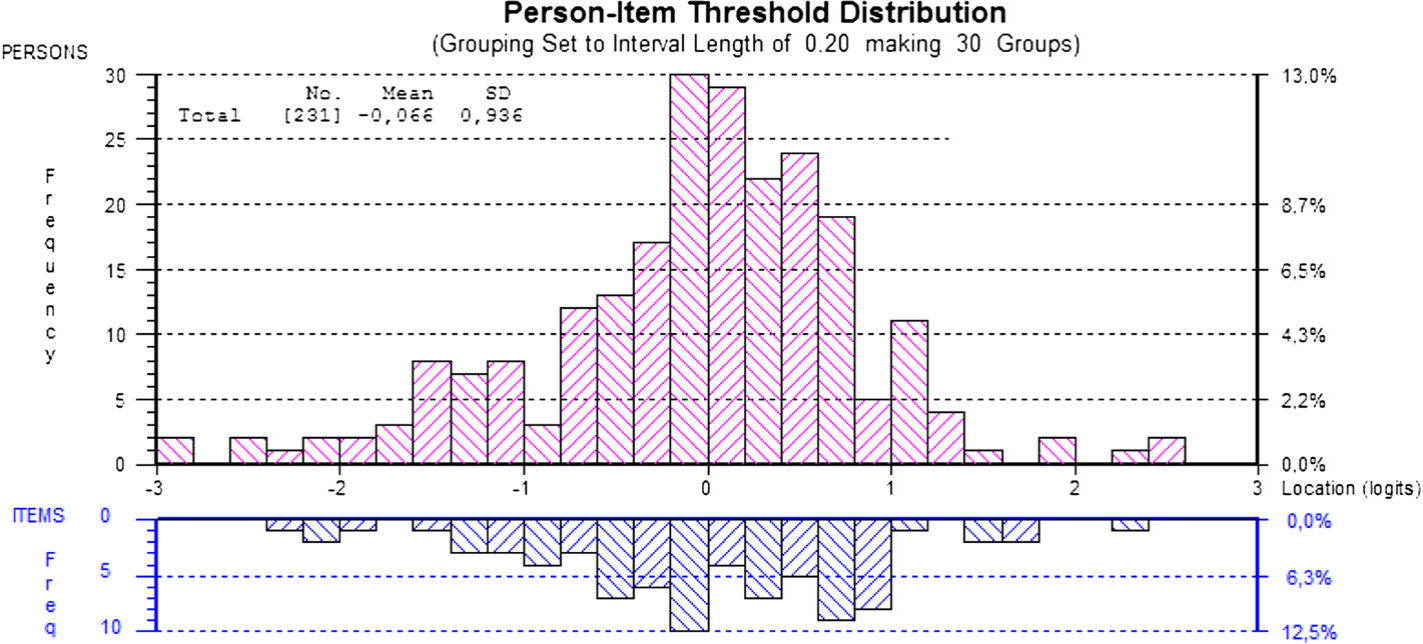
**Targeting of the MFI-20 with person – item threshold distribution (20 items).**

Targeting of the full MFI-20 scale is shown in Figure 1 with the distribution of person and item thresholds on the same logit scale. Zero on the scale denotes average severity of fatigue among the persons as well as average difficulty of the MFI-20 items. The level of fatigue severity of the MFI-20 scale was well targeted for the population of persons with post-polio syndrome.

### Test of MFI-20 subscales

In order to check subscales for fit to the Rasch model, each subscale was tested in a separate analysis, see Table 3. Tested separately four out of five subscales showed misfit to the Rasch model and local dependency was found in all subscales. To solve the issue of local dependency each subscale was analysed as a testlet, where the testlets were chosen taking into account the correlation of the residuals. In the testlet analyses, all five subscales showed fit to the Rasch model with non-significant *x*^2^ p-values (Table 3). The reliability of the subscales as expressed by the person separation index (PSI) ranged from 0.52 to 0.80. Only the Reduced motivation subscale had an unsatisfactory (PSI < 0.70) PSI value (Table 3).

### Transformation of raw scores to an interval scale

After fit to the Rasch model was achieved for the MFI-20 a transformation of the ordinal total scale scores into an interval level scale for use in parametric analyses was performed (Table 4). Dummy cases with minimum and maximum scoring were used for the transformation table to achieve interval scores between 20 and 100, which are comprehensive limits for the MFI-20 scale.

**Table 4:** **Transformation of total MFI-20 raw total score to interval scale**

**Raw score**	**Interval score**	**Raw score**	**Interval score**
20	20	61	45.1
21	27.3	62	45.9
22	32.1	63	46.6
23	35.3	64	47.3
24	37.8	65	48.0
25	39.7	66	48.7
26	41.3	67	49.4
27	42.7	68	50.2
28	43.9	69	64.6
29	45.0	70	65.0
30	46.0	71	65.5
31	46.9	72	65.9
32	47.7	73	66.3
33	48.4	74	66.7
34	49.1	75	67.1
35	49.8	76	67.6
36	50.4	77	68.0
37	51.0	78	68.5
38	51.6	79	69.0
39	52.1	80	69.4
40	52.7	81	69.9
41	53.2	82	70.5
42	53.7	83	71.0
43	54.1	84	71.6
44	54.6	85	72.2
45	55.1	86	72.8
46	55.5	87	73.4
47	55.9	88	74.1
48	56.4	89	74.9
49	56.8	90	75.7
50	37.1	91	76.6
51	37.9	92	77.6
52	38.6	93	78.7
53	39.4	94	79.9
54	40.1	95	81.4
55	40.8	96	83.2
56	41.6	97	85.4
57	42.3	98	88.4
58	43.0	99	93.0
59	43.7	100	100
60	44.4		

## Discussion

Fatigue is a common symptom and can have a substantial impact on post-polio patients. The aim of this paper was to use Rasch analysis to assess the psychometric properties of the MFI-20, a widely used questionnaire for measuring fatigue. To our knowledge, this is the first Rasch analysis of MFI-20 data in patients with post-polio syndrome.

Standardized outcome measures that effectively assess effects of interventions and treatments and/ or progression of fatigue are needed in clinical practice. Rasch analysis of existing questionnaires developed within the framework of classical test theory, such as the MFI-20, can help to assess their utility for these purposes and at the same time improve interpretability of generated scores and change in scores. The latter is particularly important for communicating clinical results to patients, community services and insurance providers.

After dealing with response dependency both the full scale and the five subscales scores of the MFI-20 subscales can be considered unidimensional. Therefore, both the total and the subscales of MFI-20 can be used to assess fatigue in persons with post-polio syndrome. This is consistent with earlier factor analytical studies of the MFI-20 [9,16,18]. Only the Reduced motivation subscale had to low reliability (PSI = 0.52) for comparisons on a group level [25]. Nonetheless, the MFI-20 comprises several items expressing the same content but worded both in a positive and in a negative way, e.g. Physically I am in *bad* condition versus Physically I am in *excellent* condition. Such items are highly correlated [32] and may be the reason for response dependency between items within the subscales.

The results show that MFI-20 meets criteria for satisfactory internal construct validity making it possible to transform raw ordinal scores into an interval metric for use in future clinical studies evaluating patients with post-polio syndrome [33]. The Rasch-based modified interval sum score is more appropriate than ordinal-based scores for use in parametric statistics to compute and compare change scores in clinical practice and research [34]. The transformation table (Table 4) can only be used to obtain transformed scores from raw scores if the respondent has filled in all items; however, several earlier studies [9,18,19] as well as our own indicate that missing item rates associated with the MFI-20 are low.

### Study limitations

Patients were recruited from all post-polio outpatient clinics in Sweden and represent a fairly homogeneous sample; hence further testing is needed in more culturally heterogeneous groups, in other languages and in other diagnosis groups. In particular, potential differential item functioning between MFI-20 language versions needs to be formally assessed and, if found, adequately dealt with to ensure the comparability of scores across countries. Item bias was only assessed in relation to gender and age; other factors potentially contributing to item bias need to be examined.

## Conclusions

Our results add to the evidence of the usefulness of the Swedish MFI-20 total scale and subscales to measure fatigue in persons with post-polio syndrome in clinical settings. The internal consistency reliability for the total score was high (PSI = 0.86), indicating that the Swedish MFI-20 scale is reliable enough to discriminate between persons and groups of persons with different levels of fatigue. The transformation table can be used to transform raw ordinal scores of the MFI-20 into interval equivalent scores for use in parametric statistical analyses in future clinical studies evaluating patients with post-polio syndrome.

## Abbreviations

DIF: Differential item functioning
MFI-20: Multidimensional fatigue inventory, an instrument to assess fatigue, with 20 items
PCA: Principal component analysis
PPS: Post-polio syndrome

## References

[1] Farbu E, Gilhus NE, Barnes MP, Borg K, de Visser M, Driessen A (2006). EFNS guideline on diagnosis and management of post-polio syndrome. Report of an EFNS task force. Eur J Neurol: Offic J Eur Fed Neurol Soc.

[2] Schanke AK, Stanghelle JK (2001). Fatigue in polio survivors. Spinal Cord.

[3] Trojan DA, Cashman NR (2005). Post-poliomyelitis syndrome. Muscle Nerve.

[4] Tersteeg IM, Koopman FS, Stolwijk-Swuste JM, Beelen A, Nollet F (2011). A 5-year longitudinal study of fatigue in patients with late-onset sequelae of poliomyelitis. Arch Phys Med Rehabil.

[5] Farbu E, Rekand T, Gilhus NE (2003). Post-polio syndrome and total health status in a prospective hospital study. Eur J Neurol: Offic J Eur Fed Neurol Soc.

[6] Trojan DA, Arnold DL, Shapiro S, Bar-Or A, Robinson A, Le Cruguel JP (2009). Fatigue in post-poliomyelitis syndrome: association with disease-related, behavioral, and psychosocial factors. PM & R : J Inj Funct Rehabil.

[7] Bruno RL, Frick NM, Cohen J (1991). Polioencephalitis, stress, and the etiology of postpolio sequelae. Orthopedics.

[8] Ostlund G, Wahlin A, Sunnerhagen KS, Borg K (2011). Post polio syndrome: fatigued patients a specific subgroup?. J Rehabil Med: Offic J UEMS Eur Board Phys Rehabil Med.

[9] Smets EM, Garssen B, Bonke B, De Haes JC (1995). The Multidimensional Fatigue Inventory (MFI) psychometric qualities of an instrument to assess fatigue. J Psychosom Res.

[10] Smets EM, Garssen B, Cull A, de Haes JC (1996). Application of the multidimensional fatigue inventory (MFI-20) in cancer patients receiving radiotherapy. Br J Cancer.

[11] Wright BD, Linacre JM (1989). Observations are always ordinal; measurements, however, must be interval. Arch Phys Med Rehabil.

[12] Gonzalez H, Sunnerhagen KS, Sjoberg I, Kaponides G, Olsson T, Borg K (2006). Intravenous immunoglobulin for post-polio syndrome: a randomised controlled trial. Lancet Neurol.

[13] MarchofDimes.: March of Dimes International Conference on Post Polio Syndrome. Identifying Best Practices in Diagnosis and Care. http://www.polioplace.org/sites/default/files/files/MOD-%20Identifying.pdf. March of Dimes, NY, USA: White Plains, 2000; pp. 9 – 11.

[14] Furst CJ, Ahsberg E (2001). Dimensions of fatigue during radiotherapy. An application of the Multidimensional Fatigue Inventory. Support Care Cancer: Offic J Multinational Assoc Support Care Cancer.

[15] Whitehead L (2009). The measurement of fatigue in chronic illness: a systematic review of unidimensional and multidimensional fatigue measures. J Pain Symptom Manage.

[16] Schwarz R, Krauss O, Hinz A (2003). Fatigue in the General Population. Onkologie.

[17] Lin JM, Brimmer DJ, Maloney EM, Nyarko E, Belue R, Reeves WC (2009). Further validation of the Multidimensional Fatigue Inventory in a US adult population sample. Popul Health Metrics.

[18] Hagelin CL, Wengstrom Y, Runesdotter S, Furst CJ (2007). The psychometric properties of the Swedish Multidimensional Fatigue Inventory MFI-20 in four different populations. Acta Oncol.

[19] Ericsson A, Mannerkorpi K (2007). Assessment of fatigue in patients with fibromyalgia and chronic widespread pain. Reliability and validity of the Swedish version of the MFI-20. Disabil Rehabil.

[20] Ericsson A, Bremell T, Mannerkorpi K (2013). Usefulness of multiple dimensions of fatigue in fibromyalgia. J Rehabil Med: Offic J UEMS Eur Board Phys Rehabil Med.

[21] Andrich D, Lyne A, Sheridan B, Luo G (2010). RUMM 2030.

[22] Gustafsson JE (1980). Testing and obtaining fit of data to the rasch model. Brit J Math Stat Psyol.

[23] Guttman LA, Stouffer SA, Guttman LA, Suchman FA, Lazarsfeld PF, Star SA, Clausen JA (1950). The basis for Scalogram analysis. Studies in social psychology in World War II, Measurement and Prediction.

[24] Rasch G (1960). Probabilistic models for some intelligence and attainment tests.

[25] Tennant A, Conaghan PG (2007). The Rasch measurement model in rheumatology: what is it and why use it? When should it be applied, and what should one look for in a Rasch paper?. Arthritis Rheum.

[26] Bland JM, Altman DG (1995). Multiple significance tests: the Bonferroni method. BMJ.

[27] Smith EV (2002). Detecting and evaluating the impact of multidimensionality using item fit statistics and principal component analysis of residuals. J Appl Meas.

[28] Teresi JA, Kleinman M, Ocepek-Welikson K (2000). Modern psychometric methods for detection of differential item functioning: application to cognitive assessment measures. Stat Med.

[29] Tennant A, Penta M, Tesio L, Grimby G, Thonnard JL, Slade A (2004). Assessing and adjusting for cross-cultural validity of impairment and activity limitation scales through differential item functioning within the framework of the Rasch model: the PRO-ESOR project. Med Care.

[30] Pallant JF, Keenan AM, Misajon R, Conaghan PG, Tennant A (2009). Measuring the impact and distress of osteoarthritis from the patients’ perspective. Health Qual Life Outcomes.

[31] Lundgren Nilsson A, Tennant A (2011). Past and present issues in Rasch analysis: the functional independence measure (FIM) revisited. J Rehabil Med: Offic J UEMS Eur Board Phys Rehabil Med.

[32] Hinz A, Michalski D, Schwarz R, Herzberg PY (2007). The acquiescence effect in responding to a questionnaire. Psycho-Soc Med.

[33] Christensen KB, Kreiner S (2010). Monte Carlo tests of the Rasch model based on scalability coefficients. Br J Math Stat Psychol.

[34] Johansson S, Kottorp A, Lee KA, Gay CL, Lerdal A (2014). Can the Fatigue Severity Scale 7-item version be used across different patient populations as a generic fatigue measure–a comparative study using a Rasch model approach. Health Qual Life Outcomes.

